# Reliability of Vertical Jump Force-Time Metrics in Collegiate Athletes Compared to Recreationally Active Individuals

**DOI:** 10.3390/life15121830

**Published:** 2025-11-28

**Authors:** Dimitrije Cabarkapa, Robert Smith, Luke Chowning, Tyler Neltner, Quincy R. Johnson, Yang Yang, Thayne A. Munce

**Affiliations:** 1 Jayhawk Athletic Performance Laboratory—Wu Tsai Human Performance Alliance, Department of Health, Sport and Exercise Sciences, University of Kansas, Lawrence, KS 66045, USA; 2 D2 Lab, 21000 Novi Sad, Serbia; 3 College of Science Technology and Health, Lindenwood University, Saint Charles, MO 63301, USA; 4 College of Education and Human Performance, Dakota State University, Madison, SD 57042, USA; 5 College of Health and Human Performance, University of Wisconsin Platteville, Platteville, WI 53818, USA

**Keywords:** asymmetries, force plate, strength, power, athlete monitoring, technology

## Abstract

As neuromuscular performance assessment has become a fundamental component of athlete monitoring, ensuring strong measurement reliability is essential for supporting accurate data-driven decision-making. Thus, the purpose of this study was twofold: (i) to examine the reliability of countermovement vertical jump (CMJ) force-time metrics obtained using a portable force plate system (Hawkin Dynamics) and (ii) to determine whether absolute and relative reliability scores differ between well-trained individuals (i.e., athletes) and those less familiar with CMJ force-plate testing (i.e., non-athletes). Seventy-four participants volunteered to take part in this investigation, of whom thirty-nine were NCAA Division-I baseball and track-and-field athletes and thirty-five age-matched non-athletes with no prior CMJ testing experience on force plates. After performing a standardized dynamic warm-up, participants performed three CMJs without arm swing while standing on a dual uniaxial force plate system sampling at 1000 Hz. Each jump trial was separated by a 30 s rest interval. Absolute and relative reliability were assessed using the coefficient of variation (CV) and intraclass correlation coefficient (ICC), respectively. The results revealed that 75% of the variables demonstrated excellent reliability. Specifically, absolute (CV < 10%) and relative (ICC > 0.750) reliability values were good to excellent for most force-time metrics of interest, including braking and propulsive phase duration, peak braking force, average propulsive power, reactive strength index-modified, countermovement depth, and jump height. In contrast, average and peak landing force and inter-limb asymmetry measures during the braking and propulsive phases displayed moderate to good reliability, whereas asymmetry-related variables during the landing phase exhibited poor reliability. In addition, athletes demonstrated lower CV and greater ICC across most metrics compared to non-athletes.

## 1. Introduction

Assessing neuromuscular performance qualities has become one of the fundamental components of the athlete monitoring process, with recent survey data showing that 91% of sports practitioners conduct daily or weekly evaluations to detect fatigue-related changes in performance [1,2,3]. Among these methods, vertical jump testing has been the most frequently employed, with the countermovement jump (CMJ) serving as the predominant assessment used by over half of sports practitioners (54%) [1,4,5]. Specifically, the CMJ represents a coordinated neuromuscular task in which an athlete lowers their center of mass to a self-selected depth, before rapidly reversing the direction of the movement to propel the body upward, until airborne. To optimize CMJ performance, one must utilize a stretch-shortening cycle in conjunction with concentric muscle contractions to produce maximal magnitudes of force as quickly as possible [6]. During the descending portion of the CMJ, muscles, tendons, and intramuscular connective tissues are rapidly lengthened, resulting in stored elastic energy, and muscle spindles are stimulated, ultimately resulting in increased motor unit recruitment [6,7]. Once the downward movement is completed, the center of mass rapidly reverses direction. During the upward phase of the CMJ, stored elastic energy is released and muscular force production increases, enabling the athlete to accelerate upward and ultimately leave the ground.

The CMJ reflects an athlete’s ability to rapidly generate and apply force during a coordinated stretch-shortening cycle, and the resulting performance metrics are widely used to evaluate fatigue, physiological readiness, and training adaptations (e.g., increased force production, faster contraction time) [2,8,9,10,11]. A substantial body of research has confirmed the CMJ as a valid and reliable measure of lower-body neuromuscular performance qualities in athletic populations, with a range of technologies now available to quantify CMJ performance, from basic jump mats to advanced force plate systems [12,13,14,15]. Specifically, a recent survey of sports practitioners in rugby union reported that 35% of CMJ assessments were performed using jump mats, likely due to their user-friendliness and cost-effectiveness [16]. However, while jump mats provide valuable performance information, they are unable to capture key mechanistic variables (i.e., ground reaction forces) that underpin jump performance. In contrast, force platforms offer a more comprehensive evaluation of neuromuscular qualities by providing a variety of force-time metrics. Although traditional systems were costly and typically embedded in laboratory floors, the emergence of portable force plates has expanded access to this technology, offering sports practitioners a valid and reliable tool for CMJ-based athlete evaluation [8,17].

Despite research consistently showing moderate to excellent reliability for key force-time variables across diverse populations (e.g., tactical personnel, physically active males, female volleyball players), these metrics can still vary as a function of training status (i.e., well-trained vs. untrained), testing protocols, equipment, sport-specific positional demands, and familiarity with the CMJ task [1,14,18]. For example, Krzyszkowski et al. [19] found that individuals with superior CMJ performance exhibited shorter time-to-takeoff and propulsive phase durations. Similarly, Harry et al. [20] reported that individuals with superior CMJ performance capabilities completed the unloading phase more quickly than their lower-performing counterparts, while propulsive phase duration has been identified as a negative predictor of jump height [21]. Taken together, these findings indicate that individuals capable of generating greater eccentric force (i.e., storing more elastic energy) and transitioning quickly through the concentric phase of the movement tend to exhibit superior CMJ performance, highlighting the importance of technical proficiency and task familiarity in influencing jump outcomes and the reliability of force-time metrics.

While force plates are becoming increasingly used to assess lower-body neuromuscular performance qualities, the performance testing typically involves one to three CMJs within a single testing session [10,22]. While reliability has been established in various populations, a direct comparison of reliability between athletes and non-athletes using the same equipment is lacking, particularly given that participants may range from well-trained athletes accustomed to CMJ testing to individuals with limited experience performing CMJ tasks on portable force plates, representing the gap this study aims to address. Therefore, the purpose of the present study was twofold: (i) to examine the reliability of CMJ force-time metrics obtained using a dual uniaxial force plate system (i.e., Hawkin Dynamics), and (ii) to determine whether absolute and relative reliability scores differ between well-trained individuals (i.e., athletes) and those less familiar with CMJ force-plate testing (i.e., non-athletes). It was hypothesized that CMJ-related performance parameters would demonstrate high test–retest reliability, with athletes exhibiting greater absolute and relative reliability scores compared to non-athletes due to the potential influence of a learning effect.

## 2. Materials and Methods

### 2.1. Participants

A total of seventy-four male (n = 36) and female (n = 38) participants volunteered to take part in this investigation. Among them, thirty-nine were National Athletic Collegiate Association (NCAA) Division-I baseball (n = 16) and track-and-field (n = 23) athletes (age = 20.9 ± 2.2 years; body mass = 74.5 ± 12.3 kg), while thirty-five were non-athletes (i.e., recreationally active college-aged individuals; age = 21.3 ± 3.0 years; body mass = 79.2 ± 12.4 kg). As part of their regular training programs, the collegiate athletes consistently engaged in organized team practices, including resistance-training sessions supervised by their respective strength and conditioning staff. They were also accustomed to CMJ assessments, which were performed on a weekly basis as part of their ongoing performance monitoring routines. In contrast, the non-athletes had no prior experience performing CMJ assessments and reported no structured familiarity with countermovement jump technique or force-plate testing. All participants were free from musculoskeletal injuries that could restrict or impair jumping performance. The testing procedures were reviewed and approved by the University’s Institutional Review Board, and written informed consent was obtained from all participants prior to data collection.

### 2.2. Procedures

Each participant completed a standardized dynamic warm-up that included movements such as A-skips, butt kicks, and side-to-side lunges. Following the warm-up procedure, athletes performed three CMJs with their hands akimbo (i.e., maintaining their hands on their hips throughout the entire motion) on a dual uniaxial force plate system sampling at 1000 Hz (Hawkin Dynamics, Model 0484; Westbrook, ME, USA). Participants were instructed to descend rapidly to a self-selected depth and then explosively extend the lower limbs to produce a maximum-effort vertical jump (i.e., focus on pushing the ground as explosively as possible), aiming to attain the highest jump height. Prior to each attempt, participants placed their feet parallel and centered on the force plates, which were positioned with a 2 cm gap between them, and were instructed to land in the same spot to ensure consistent takeoff and landing mechanics. A 30-second rest period was provided between trials to minimize the potential fatigue effects, and the testing was conducted approximately 24 h after the last training session. Strong verbal encouragement was provided by research staff throughout the data collection process.

The variables analyzed in this study were selected based on prior literature identifying force-time metrics with strong practical relevance within braking (i.e., eccentric), propulsive (i.e., concentric), and landing phases of the CMJ [16,23,24,25,26]. Time-to-takeoff (i.e., contraction time) was defined as the period from movement initiation to the instant of take-off. Following manufacturer recommendations, the braking phase was identified as the interval from the point of minimum ground reaction force until vertical velocity reached zero, while the propulsive phase encompassed the period from zero velocity to takeoff. The landing phase began at the instant of touchdown and ended when the center of mass velocity reached zero for the first time. Countermovement depth was quantified as the peak negative vertical displacement of the system’s center of mass. In addition, the absolute inter-limb asymmetry percentage was calculated for each force-related metric (i.e., [L_leg_ − R_leg_/(L_leg_ + R_leg_/2)] × 100), within braking, propulsive, and landing phases of the jumping motion [23]. For context, several commonly reported CMJ variables include peak braking force, which is widely interpreted as an indicator of eccentric strength; reactive strength index (RSI)-modified, which reflects stretch-shortening cycle efficiency; countermovement depth, which describes the athlete’s movement strategy; and contraction time, which indicates how quickly the athlete can generate force and transition through the jump, each representing metrics of practical significance in applied sport settings. The detailed definition of each force-time metric can be found at *www.hawkindynamics.com* (accessed on 26 November 2025) [12,16].

### 2.3. Statistical Analysis

Descriptive statistics, means, and standard deviations ($\bar{x}$ ± SD), were calculated for each dependent variable (Trial 1–3). Absolute reliability was assessed using the coefficient of variation (CV), calculated as the standard deviation divided by the mean across repeated trials and expressed as a percentage. Corresponding 95% confidence intervals (CI) were also computed, with CV values < 10% considered acceptable [15,27]. The standard error of measurement (SEM) and minimal detectable change (MDC) were calculated for some of the key force-time metrics. In addition, the intraclass correlation coefficient (ICC_3,k_) and its associated 95% CI were calculated to assess relative agreement for each force-time metric, overall and within each group individually (i.e., athletes vs. non-athletes), and interpreted based on the lower bound of 95% CI as <0.50 poor; 0.50–0.74 moderate; 0.75–0.90 good; >0.90 excellent [28]. All statistical analyses were completed with SPSS (Version 29.0; IBM Corp., Armonk, NY, USA).

## 3. Results

Descriptive statistics, means and standard deviations ($\bar{x}$ ± SD), for each dependent variable are presented in Table 1. Overall, 75% of the variables demonstrated excellent reliability scores. Specifically, absolute (CV < 10%) and relative (ICC > 0.750) reliability were deemed good to excellent for the majority of force-time metrics, excluding average and peak landing forces and inter-limb asymmetry measures during both the braking and propulsive phases of the jumping motion, which exhibited moderate to good reliability (Table 2 and Table 3). The SEM and MDC, respectively, for some of the key force-time metrics were the following (overall): braking phase duration (0.04 and 0.13 s), propulsive phase duration (0.06 and 0.18 s), average braking power (84.0 and 233 W), average propulsive power (73 and 202 W), jump height (0.91 and 1.98 cm), countermovement depth (1.73 and 4.8 cm) RSI-modified (0.03 and 0.06). In contrast, asymmetry-related variables during the landing phase of the jumping motion revealed poor reliability scores, with CV values ranging from 61.60 to 70.13% and ICC values ranging from 0.285 to 0.498. For instance, jump height demonstrated excellent reliability (CV = 3.01% and ICC = 0.994), while peak landing force asymmetry demonstrated poor reliability (CV = 69.87% and ICC = 0.290). In addition, athletes tended to demonstrate lower variability and greater reliability across most metrics compared to non-athletes. An exception to this pattern occurred in the landing phase, where athletes exhibited higher CV values and lower ICC relative to non-athletes, indicating greater variability during this phase of the movement (Table 2 and Table 3).

## 4. Discussion

The purpose of the present study was to evaluate the reliability of force-time metrics within the braking, propulsive, and landing phases of the CMJ, as well as to compare absolute and relative reliability indices (i.e., CV and ICC) between well-trained individuals (i.e., athletes) and their age-matched counterparts (i.e., non-athletes) with limited familiarity performing CMJs on force plates. To our knowledge, while prior work has explored the reliability of selected CMJ force-time metrics, the present study is the first to include asymmetry-related measures across all movement phases, including the landing phase of CMJ, and to directly compare reliability profiles between athletes and non-athletes. Overall, the results revealed that 75% of the variables examined demonstrated excellent levels of reliability. Specifically, absolute (CV < 10%) and relative (ICC > 0.750) reliability were classified as good to excellent for most force-time metrics of interest, including braking and propulsive phases duration, peak braking force, average propulsive power, RSI-modified, countermovement depth, and jump height. In contrast, average and peak landing forces and inter-limb asymmetry measures during the braking and propulsive phases displayed moderate to good reliability, whereas asymmetry-related variables during the landing phase exhibited poor reliability, indicating substantial measurement variability across repeated jump trials. In addition, athletes tended to demonstrate lower variability and greater reliability across most metrics compared to non-athletes. Yet, an exception to this pattern occurred within the landing phase of CMJ, where athletes exhibited higher CV values and lower ICC relative to non-athletes, indicating greater variability during this phase of the movement.

Previous studies have examined the reliability of force-time characteristics within both the braking and propulsive phases of CMJ [1,15,29,30]. For instance, when studying a cohort of 112 athletes from a variety of individual and team sports, Merrigan et al. [15] reported excellent absolute and relative reliability scores for key force-time metrics such as braking and propulsive average force and peak power (CV = 0.06–9.24 and ICC = 0.930–0.990). Similarly, high reliability has been demonstrated for RSI-modified (CV = 5.81 and ICC = 0.910) and jump height (CV = 3.20 and ICC = 0.970), which closely aligns with the results obtained in the present investigation, further supporting the robustness of these outcome measures [15]. Nevertheless, Heishman et al. [30] reported lower reliability values for several CMJ force-time metrics in NCAA Division-I male basketball players compared with those observed in the present athlete cohort, such as RSI-modified (CV = 9.1 and ICC = 0.879). While further research is warranted on this topic, these discrepancies may be partly attributed to sport-specific movement characteristics, as basketball athletes frequently perform reactive, multidirectional, and context-dependent jumps that may induce greater variability in force-producing strategies [31]. In contrast, athletes from more mechanically constrained sports (e.g., track and field) typically execute standardized and technically consistent movements, leading to more stable force-time profiles across repeated trials. Moreover, it should be noted that the same group of authors found lower inter-session reliability scores (absolute and relative) during CMJ with arm swing compared with the no-arm-swing condition [30]. While incorporating an arm swing enhances force and power output, accounting for approximately 31.5% of peak ground-reaction force, it may simultaneously introduce greater movement variability due to increased coordination demands associated with upper- and lower-body synchronization [32,33].

In contrast to the braking and propulsive phases, the present study found that substantially lower absolute and relative reliability values were observed for the landing phase and for asymmetry-related metrics. Consistent with this, prior research has demonstrated that inter-limb asymmetry and landing-mechanics variables generally show greater variability and reduced test–retest stability relative to CMJ performance markers such as peak braking force, average propulsive power, and countermovement depth [34,35,36]. In the current investigation, asymmetry magnitudes ranged from approximately 4.4–5.6% during the braking phase and 2.7–3.7% during the propulsive phase but increased substantially during the landing phase (10.2–13.0%). These findings align with the recent work of Cone et al. [37], who observed greater asymmetry in ground reaction force distribution during landing compared with the take-off phase of the CMJ. Also, it should be noted that the aforementioned asymmetry magnitudes fall within previously proposed thresholds considered potentially meaningful for monitoring performance and injury risk profiles in athletes [23,34]. Moreover, from a biomechanical perspective, landing is a highly demanding neuromuscular task that differs fundamentally from upward propulsion. Unlike force production during the propulsive phase of the jumping motion, successful landing requires rapid modulation of lower-limb stiffness, proprioceptive feedback, and neuromuscular control, and pre-landing kinematic positioning to attenuate impact forces and reestablish postural stability [38,39,40]. As a result, landing mechanics are inherently more variable and sensitive to fluctuations in fatigue, attentional state, and inter-joint coordination strategies. Furthermore, asymmetry indices can appear disproportionately large when baseline bilateral differences are small, which mathematically amplifies variability and may reduce reliability across repeated sessions [35]. Collectively, these factors help explain the poorer reproducibility of landing phase and asymmetry-based CMJ metrics observed in the present study and highlight the need for caution when interpreting single-session asymmetry data, particularly in well-trained athletes who typically exhibit minimal inter-limb differences. Additionally, given that highly trained athletes generally achieve greater jump heights, the associated increases in eccentric loading during landing may further accentuate variability in asymmetry measures due to higher mechanical and neuromuscular demands upon ground contact. Moreover, it should be noted that larger variability in landing kinetics among well-trained athletes may in fact be advantageous, as functional movement variability can help distribute loads across tissues and reduce overuse injury risk, whereas excessively constrained or excessively erratic movement patterns have each been linked to elevated injury susceptibility, underscoring the importance of an optimal level of variability for maintaining neuromuscular resilience [41,42].

Another important consideration in the present study relates to the observed differences in the reliability of force-time metrics across the braking and propulsive phases of the CMJ. In line with the current findings, athletes (i.e., well-trained individuals) generally demonstrated lower CV and higher ICC across repeated jump trials, reflecting greater absolute and relative reliability. Although standardized verbal cues were provided across all participants (e.g., “focus on pushing the ground as explosively as possible”), the potential influence of a learning effect needs to be acknowledged. Previous research has documented meaningful improvements in various performance-related tasks due to familiarization when conducting tests such as the one-repetition-maximum bench press and back squat, as well as the Wingate anaerobic test [43,44]. For example, Dias et al. [44] reported progressive increases in maximal strength of 2.4%, 3.4%, and 5.4% from the first to final testing sessions, while Barefield et al. [43] demonstrated higher peak and average power outputs from the initial Wingate trial (604.9–764.5 W) to the final trial (634.7–867.6 W), indicating a clear learning effect with no physiological changes. More recently, Cabarkapa et al. [45] showed that CMJ force-time metrics, including both performance outcomes (e.g., jump height) and movement strategy variables (e.g., countermovement depth), remained consistent across five consecutive testing sessions in physically active individuals familiar with jumping tasks. Considering that athletes in the present investigation routinely engaged in structured strength and conditioning programming and regularly performed CMJ assessments on force plates, it is likely that this familiarization, in combination with consistent verbal cueing, likely contributed to the high reliability values observed, which were comparable to those reported by Cabarkapa et al. [45]. Therefore, to optimize the reliability of CMJ-derived force-time measurements, practitioners should ensure adequate familiarization with the testing protocol, particularly when introducing new technologies such as portable force plates, and apply consistent movement cues to promote stable and repeatable performance outputs.

While this study offers valuable insight into the reliability of commonly utilized CMJ force-time metrics for assessing lower-body neuromuscular performance across braking, propulsive, and landing phases of the CMJ, several limitations should be acknowledged. First, the athlete cohort consisted of collegiate athletes, which may limit the generalizability of the findings to youth or professional athletic populations who may exhibit different neuromuscular and technical profiles. Second, the present investigation focused solely on the CMJ with no arm swing (i.e., hands akimbo); therefore, reliability outcomes may differ when examining other jump modalities, such as the drop jump or squat jump, as well as CMJ with arm swing. Third, only intra-session reliability was assessed, and future research should focus on evaluating inter-session reliability (e.g., multiple days) to determine the temporal stability of these metrics. Additionally, reliability under conditions of neuromuscular fatigue remains unclear, and examining how fatigue influences both force-time and asymmetry outcomes represents an important direction for future research. Moreover, future studies should incorporate kinematic analyses through markerless motion-capture systems or advanced video-based methods to evaluate movement-strategy variables (e.g., segmental coordination, kinematic sequencing) and determine the reliability of these biomechanical parameters alongside force-time metrics.

## 5. Conclusions

In conclusion, the present findings of the present study indicate that 75% of the examined CMJ force-time metrics demonstrated excellent reliability, including braking and propulsive phase duration, peak braking force, average propulsive power, RSI-modified, braking and propulsive average and peak velocities, countermovement depth, and jump height (CV < 10% and ICC > 0.750). In contrast, average and peak landing forces and inter-limb asymmetry measures during braking and propulsion showed only moderate to good reliability, while landing-phase asymmetry metrics exhibited poor reproducibility. Notably, athletes displayed more stable force-time profiles and lower variability than non-athletes across most variables. However, this trend reversed in the landing phase, where athletes demonstrated greater variability, likely due to higher eccentric demands associated with greater jump performance. Given these inconsistencies, force-time metrics with lower reproducibility should be interpreted cautiously, and practitioners may need multiple trials or repeated testing sessions to establish a dependable baseline before drawing meaningful conclusions regarding an athlete’s performance capabilities. Overall, this study indicates that CMJ performed on a portable force plate system (Hawkin Dynamics) is a reliable tool for assessing lower-limb neuromuscular performance characteristics in collegiate athletes.

## Figures and Tables

**Table 1:** Descriptive statistics, means and standard deviations, for each force-time metric (overall).

Variable [Unit]	Trial 1	Trial 2	Trial 3
*Braking phase*			
Braking phase duration [s]	0.161 ± 0.034	0.160 ± 0.034	0.161 ± 0.035
Average braking force [N]	1444.2 ± 315.9	1430.8 ± 309.2	1385.5 ± 299.8
Peak braking force [N]	1935.7 ± 458.9	1922.1 ± 454.4	1878.2 ± 432.1
Average braking velocity [m·s^−1^]	0.885 ± 0.151	0.864 ± 0.143	0.821 ± 0.148
Peak braking velocity [m·s^−1^]	1.362 ± 0.244	1.328 ± 0.236	1.250 ± 0.239
Average braking power [W]	1161.9 ± 383.1	1122.9 ± 355.7	1034.1 ± 345.9
Peak braking power [W]	1513.9 ± 504.4	1389.4 ± 501.9	1389.4 ± 501.9
Braking impulse [N·s]	229.7 ± 60.4	226.0 ± 57.8	221.3 ± 58.7
Braking net impulse [N·s]	105.9 ± 29.1	102.9 ± 27.5	97.0 ± 26.8
Average braking force asymmetry [%]	5.4 ± 4.9	5.3 ± 4.8	5.6 ± 4.6
Peak braking force asymmetry [%]	4.8 ± 4.4	4.4 ± 4.3	4.6 ± 4.5
*Propulsive phase*			
Propulsive phase duration [s]	0.259 ± 0.037	0.256 ± 0.039	0.251 ± 0.038
Average propulsive force [N]	1603.4 ± 341.7	1611.8 ± 351.8	1615.9 ± 346.1
Peak propulsive force [N]	1977.1 ± 455.5	1975.0 ± 458.7	1965.9 ± 448.2
Average propulsive velocity [m·s^−1^]	1.654 ± 0.202	1.643 ± 0.193	1.622 ± 0.194
Peak propulsive velocity [m·s^−1^]	2.843 ± 0.347	2.834 ± 0.332	2.810 ± 0.341
Average propulsive power [W]	2449.1 ± 717.5	2445.6 ± 711.4	2418.9 ± 695.9
Peak propulsive power [W]	4315.9 ± 1208.6	4330.2 ± 1197.7	4308.6 ± 1202.9
Propulsive impulse [N·s]	412.3 ± 93.5	409.5 ± 90.5	403.3 ± 91.8
Propulsive net impulse [N·s]	215.1 ± 53.3	214.3 ± 51.7	211.6 ± 51.5
Average propulsive force asymmetry [%]	2.7 ± 2.5	2.8 ± 2.2	2.7 ± 2.4
Peak propulsive force asymmetry [%]	3.7 ± 3.2	3.4 ± 2.9	2.9 ± 2.6
*Landing phase*			
Average landing force [N]	951.7 ± 220.9	955.4 ± 213.5	928.2 ± 236.6
Peak landing force [N]	4149.5 ± 1694.6	4331.0 ± 1782.5	4474.1 ± 1835.1
Average landing force asymmetry [%]	11.9 ± 11.1	12.0 ± 11.9	13.0 ± 10.1
Peak landing force asymmetry [%]	12.5 ± 12.3	11.7 ± 11.6	10.2 ± 7.9
*Other*			
Jump height [cm]	38.6 ± 9.9	38.4 ± 9.4	37.6 ± 9.7
Jump momentum [kg·m·s^−1^]	213.8 ± 53.1	212.9 ± 51.4	210.4 ± 51.2
Countermovement depth [cm]	32.5 ± 7.1	31.8 ± 7.4	30.5 ± 7.4
Stiffness [N·m^−1^]	6165.6 ± 1845.2	6365.6 ± 2216.7	6476.1 ± 2284.6
Force at minimum displacement [N]	1929.3 ± 459.1	1916.2 ± 460.0	1871.8 ± 429.7
RSI-modified [ratio]	0.525 ± 0.137	0.531 ± 0.139	0.514 ± 0.132

*Note: RSI—reactive strength index.*

**Table 2:** Coefficient of variation (CV) and 95% confidence interval (CI) for each force-time metric, within each group and overall.

Variable [Unit]	Athletes	Non-Athletes	Overall
*Braking Phase*			
Braking phase duration [s]	5.36 [4.50–6.23]	7.72 [6.91–8.63]	6.51 [5.65–7.37]
Average braking force [N]	4.12 [3.58–4.67]	4.83 [4.28–5.37]	4.46 [3.91–5.00]
Peak braking force [N]	3.09 [2.61–3.59]	4.31 [3.80–4.79]	3.69 [3.20–4.19]
Average braking velocity [m·s^−1^]	4.87 [4.15–5.59]	6.45 [5.73–7.17]	5.58 [4.86–6.30]
Peak braking velocity [m·s^−1^]	5.80 [5.03–6.77]	6.91 [5.97–7.66]	6.33 [5.50–7.17]
Average braking power [W]	8.18 [7.06–9.32]	9.64 [8.51–10.8]	8.81 [7.69–9.95]
Peak braking power [W]	7.48 [6.61–8.36]	9.98 [9.10–10.86]	8.55 [7.57–9.53]
Braking impulse [N·s]	3.55 [2.93–4.24]	4.43 [3.80–5.08]	3.96 [3.31–4.59]
Braking net impulse [N·s]	5.88 [5.09–6.79]	6.87 [6.02–7.71]	6.37 [5.52–7.22]
Average braking force asymmetry [%]	61.72 [54.01–69.42]	54.37 [46.67–62.07]	59.46 [51.75–67.16]
Peak braking force asymmetry [%]	64.31 [56.48–72.14]	56.49 [48.67–64.32]	61.68 [53.85–69.51]
*Propulsive phase*			
Propulsive phase duration [s]	3.55 [2.98–4.13]	4.65 [4.08–5.22]	4.06 [3.48–4.63]
Average propulsive force [N]	1.62 [1.37–1.88]	2.48 [2.12–2.65]	2.01 [1.75–2.27]
Peak propulsive force [N]	2.52 [2.09–2.91]	3.53 [3.11–3.95]	3.00 [2.59–3.43]
Average propulsive velocity [m·s^−1^]	1.59 [1.34–1.86]	2.14 [1.86–2.39]	1.90 [1.63–2.17]
Peak propulsive velocity [m·s^−1^]	0.98 [0.76–1.21]	1.74 [1.41–1.88]	1.28 [1.05–1.51]
Average propulsive power [W]	1.93 [1.57–2.28]	3.24 [2.91–3.58]	2.56 [2.22–2.90]
Peak propulsive power [W]	1.52 [1.32–1.75]	2.10 [1.82–2.23]	1.76 [1.56–1.96]
Propulsive impulse [N·s]	1.95 [1.57–2.33]	2.67 [2.19–2.96]	2.25 [1.87–2.63]
Propulsive net impulse [N·s]	1.06 [0.79–1.33]	1.95 [1.67–2.22]	1.48 [1.20–1.72]
Average propulsive force asymmetry [%]	53.92 [47.38–60.45]	56.84 [50.30–63.38]	56.03 [49.49–62.56]
Peak propulsive force asymmetry [%]	67.99 [59.73–76.24]	49.68 [41.42–57.93]	60.55 [52.29–68.80]
*Landing phase*			
Average landing force [N]	11.82 [8.98–14.66]	8.44 [5.60–11.28]	10.35 [7.51–13.19]
Peak landing force [N]	13.03 [11.06–15.01]	13.72 [11.75–15.71]	13.21 [11.25–15.20]
Average landing force asymmetry [%]	63.58 [57.33–69.83]	61.60 [55.35–67.85]	64.36 [58.11–70.61]
Peak landing force asymmetry [%]	70.13 [62.33–77.92]	67.10 [59.30–74.89]	69.07 [61.27–76.87]
*Other*			
Jump height [cm]	2.17 [1.67–2.66]	3.90 [3.12–4.87]	3.01 [2.35–3.67]
Jump momentum [kg·m·s^−1^]	1.05 [0.82–1.29]	1.98 [1.56–2.45]	1.50 [1.16–1.83]
Countermovement depth [cm]	4.63 [3.68–5.77]	6.63 [5.17–8.09]	5.8 [4.40–6.92]
Stiffness [N·m^−1^]	5.20 [4.04–6.37]	7.99 [6.82–9.14]	6.51 [5.35–7.67]
Force at minimum displacement [N]	3.15 [2.61–3.57]	4.31 [3.80–4.79]	3.70 [3.21–4.20]
RSI-modified [ratio]	4.89 [4.20–5.75]	6.27 [5.49–7.04]	5.52 [4.74–6.29]

*Note: RSI—reactive strength index.*

**Table 3:** Intraclass correlation coefficient (ICC) and the associated 95% confidence intervals (CI) for each force-time metric.

Variable [Unit]	Athletes	Non-Athletes	Overall
*Braking phase*			
Braking phase duration [s]	0.938 [0.901–0.966]	0.893 [0.824–0.939]	0.915 [0.864–0.950]
Average braking force [N]	0.983 [0.969–0.991]	0.977 [0.955–0.988]	0.980 [0.967–0.988]
Peak braking force [N]	0.992 [0.986–0.995]	0.984 [0.970–0.992]	0.988 [0.981–0.992]
Average braking velocity [m·s^−1^]	0.921 [0.792–0.975]	0.883 [0.759–0.975]	0.900 [0.775–0.961]
Peak braking velocity [m·s^−1^]	0.925 [0.801–0.982]	0.885 [0.751–0.922]	0.908 [0.752–0.949]
Average braking power [W]	0.962 [0.906–0.982]	0.911 [0.801–0.957]	0.941 [0.834–0.977]
Peak braking power [W]	0.938 [0.870–0.969]	0.893 [0.790–0.945]	0.913 [0.821–0.955]
Braking impulse [N·s]	0.988 [0.974–0.994]	0.973 [0.953–0.985]	0.979 [0.962–0.992]
Braking net impulse [N·s]	0.972 [0.919–0.988]	0.934 [0.846–0.969]	0.950 [0.862–0.971]
Average braking force asymmetry [%]	0.870 [0.782–0.925]	0.795 [0.647–0.888]	0.844 [0.773–0.875]
Peak braking force asymmetry [%]	0.809 [0.680–0.891]	0.857 [0.754–0.921]	0.841 [0.769–0.894]
*Propulsive phase*			
Propulsive phase duration [s]	0.960 [0.922–0.979]	0.908 [0.849–0.949]	0.938 [0.862–0.966]
Average propulsive force [N]	0.997 [0.995–0.999]	0.993 [0.990–0.996]	0.996 [0.993–0.997]
Peak propulsive force [N]	0.994 [0.989–0.996]	0.965 [0.940–0.980]	0.979 [0.965–0.992]
Average propulsive velocity [m·s^−1^]	0.990 [0.980–0.995]	0.987 [0.974–0.993]	0.989 [0.980–0.993]
Peak propulsive velocity [m·s^−1^]	0.988 [0.972–0.994]	0.977 [0.952–0.989]	0.983 [0.969–0.990]
Average propulsive power [W]	0.995 [0.992–0.997]	0.979 [0.964–0.989]	0.989 [0.984–0.993]
Peak propulsive power [W]	0.997 [0.995–0.998]	0.993 [0.998–0.996]	0.995 [0.993–0.997]
Propulsive impulse [N·s]	0.995 [0.989–0.998]	0.985 [0.973–0.992]	0.989 [0.977–0.996]
Propulsive net impulse [N·s]	0.997 [0.994–0.999]	0.990 [0.980–0.995]	0.993 [0.989–0.996]
Average propulsive force asymmetry [%]	0.714 [0.525–0.839]	0.722 [0.574–0.835]	0.717 [0.532–0.829]
Peak propulsive force asymmetry [%]	0.653 [0.510–0.797]	0.695 [0.541–0.816]	0.669 [0.503–0.800]
*Landing phase*			
Average landing force [N]	0.703 [0.509–0.830]	0.726 [0.583–0.836]	0.697 [0.501–0.819]
Peak landing force [N]	0.825 [0.729–0.895]	0.810 [0.701–0.889]	0.817 [0.748–0.873]
Average landing force asymmetry [%]	0.349 [0.102–0.621]	0.498 [0.144–0.723]	0.408 [0.138–0.604]
Peak landing force asymmetry [%]	0.292 [0.189–0.597]	0.285 [0.237–0.609]	0.290 [0.093–0.523]
*Other*			
Jump height [cm]	0.996 [0.991–0.998]	0.990 [0.980–0.995]	0.994 [0.989–0.996]
Jump momentum [kg·m·s^−1^]	0.997 [0.994–0.999]	0.990 [0.979–0.995]	0.993 [0.989–0.996]
Countermovement depth [cm]	0.952 [0.882–0.978]	0.938 [0.891–0.966]	0.945 [0.887–0.971]
Stiffness [N·m^−1^]	0.979 [0.964–0.988]	0.954 [0.920–0.974]	0.967 [0.951–0.978]
Force at minimum displacement [N]	0.992 [0.986–0.995]	0.984 [0.971–0.991]	0.988 [0.982–0.992]
RSI-modified [ratio]	0.981 [0.969–0.989]	0.965 [0.937–0.981]	0.977 [0.965–0.985]

*Note: RSI—reactive strength index.*

## Data Availability

Due to Institutional Review Board rules and regulations, the data is not publicly available.
